# Acute Tubulointerstitial Nephritis in Clinical Oncology: A Comprehensive Review

**DOI:** 10.3390/ijms22052326

**Published:** 2021-02-26

**Authors:** Laura Martínez-Valenzuela, Juliana Draibe, Xavier Fulladosa, Montserrat Gomà, Francisco Gómez, Paula Antón, Josep María Cruzado, Joan Torras

**Affiliations:** 1 Nephrology Department, Bellvitge University Hospital, Hospitalet de Llobregat, 08907 Barcelona, Spain; lmartinezv@bellvitgehospital.cat (L.M.-V.); xfulladosa@bellvitgehospital.cat (X.F.); fgomezp@bellvitgehospital.cat (F.G.); panton@bellvitgehospital.cat (P.A.); jmcruzado@bellvtigehospital.cat (J.M.C.); 2 IDIBELL Biomedical Research Institute, Hospitalet de Llobregat, 08907 Barcelona, Spain; 3 Clinical Science Department, Campus de Bellvitge, Barcelona University, L’Hospitalet de Llobregat, 08907 Barcelona, Spain; 4 Pathology Department, Bellvitge University Hospital, Hospitalet de Llobregat, 08907 Barcelona, Spain; mgoma@bellvitgehospital.cat

**Keywords:** acute tubulointerstitial nephritis, onconephrology, acute kidney injury

## Abstract

Acute kidney injury in patients who suffer a malignancy is a common complication. Due to its high prevalence and effective treatment, one of the most frequent causes that both oncologists and nephrologists must be aware of is acute tubulointerstitial nephritis (ATIN). ATIN is an immunomediated condition and the hallmark of the disease, with the presence of a tubulointerstitial inflammatory infiltrate in the renal parenchyma. This infiltrate is composed mainly of T lymphocytes that can be accompanied by macrophages, neutrophils, or eosinophils among other cells. One of the major causes is drug-related ATIN, and some antineoplastic treatments have been related to this condition. Worthy of note are the novel immunotherapy treatments aimed at enhancing natural immunity in order to defeat cancer cells. In the context of the immunosuppression status affecting ATIN patients, some pathogen antigens can trigger the development of the disease. Finally, hematological malignancies can also manifest in the kidney leading to ATIN, even at the debut of the disease. In this review, we aim to comprehensively examine differential diagnosis of ATIN in the setting of a neoplastic patient.

## 1. Introduction 

Acute tubulointerstitial nephritis (ATIN) is a frequent cause of acute kidney injury (AKI). Its prevalence ranges from 15 to 20% of AKI cases. It is an immune-mediated disease with a prominent participation of cellular immunity, especially T-lymphocytes [1]. It is frequently associated with therapy with certain drugs, which can act as a planted antigen in the kidney or as a hapten that can become nephritogenic. There are other causes of ATIN other than drugs, including systemic autoimmune diseases and various infectious agents, among others. In some cases, ATIN can be idiopathic [2]. Drugs trigger a type IV delayed hypersensitivity reaction that leads to renal parenchyma infiltration by immune cells [3].

In the area of oncology, the relationship between kidney disease and cancer may be bidirectional. On one side, neoplastic patients can present with AKI secondary to anticancer drug-associated nephrotoxicity. According to current expertise, nephrotoxicity can be associated with a wide range of underlying mechanisms, such as acute tubular necrosis (ATN), thrombotic microangiopathy (TMA), nephrotic syndrome of diverse etiologies, and also ATIN [4]. On the other hand, kidney failure can limit the therapeutic arsenal available for cancer treatment due to impaired excretion of drugs. Decreased glomerular filtration rate may be a contraindication for some oncological therapies. 

Neoplastic patients may have specific clinical conditions. They are more prone to developing atypical infections with a more severe clinical course due to immunosuppression associated with cancer itself or with antineoplastic treatments [5]. Clinicians must be aware of this when facing a cancer patient with AKI and especially when ATIN is present. Finally, ATIN can be the first manifestation of a hematological malignancy or can appear in a previously diagnosed patient. 

Irrespective of the clinical presentation, kidney biopsy is the gold standard diagnostic test for both entities, and specific staining techniques or in situ hybridization can help to find the causal agent. Regarding conventional ATIN, kidney biopsy reveals the presence of tubulointerstitial infiltrates composed mainly of lymphocytes, but plasma cells, macrophages, or eosinophils can also be present [6]. When viruses are in the ATIN picture, cytopathic changes in tubular cells can be observed by light microscopy. Immunohistochemistry evaluation of the kidney tissue is key to identifying the cellular profile of the infiltrates. Nucleic acid tests in kidney tissue can identify the presence of certain pathogens or mutations associated with hematological diseases.

In this review, we aim to summarize the different causes of ATIN in the oncological population with the focus on its pathophysiology. 

## 2. Drug-Related ATIN 

Oncological patients are frequently polymedicated. They receive specific anticancer therapy, together with a myriad of drugs to treat symptoms or complications associated with neoplasms. Some of these drugs, such as non-steroidal anti-inflammatory drugs (NSAIDs) [7], proton pump inhibitors [8] (PPIs), or antibiotics [9,10], often used in combination, have been described as a trigger for the development of ATIN.

**Figure 1:**
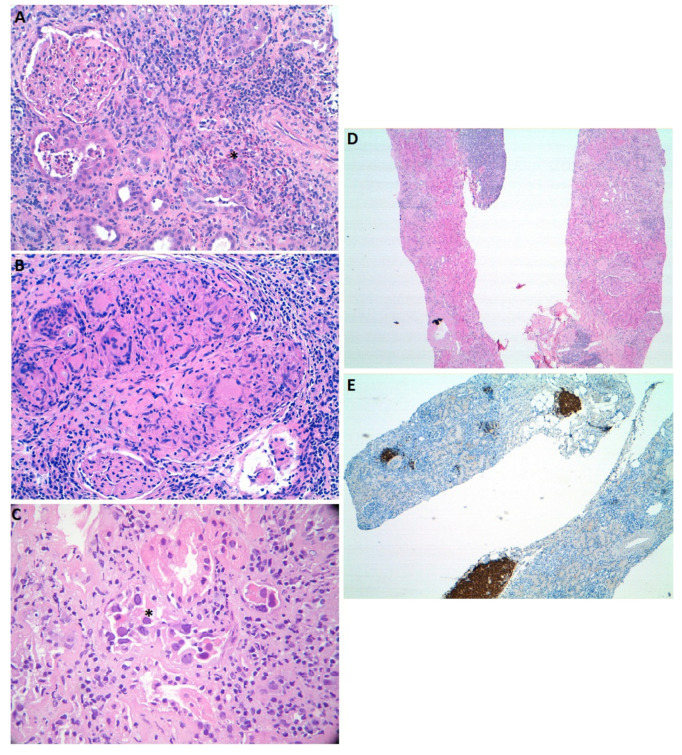
Light microscopy images illustrating acute interstitial nephritis cases with characteristic features. Panel (**A**). Extensive interstitial inflammatory infiltrates in a case of drug-induced acute tubulointerstitial nephritis. Eosinophils associated with peritubular infiltrates (*) (hematoxylin and eosin, 200×). Panel (**B**). Acute tubulointerstitial nephritis associated with Bacillus Calmette–Guerin treatment. Interstitial necrotizing granuloma (hematoxylin and eosin, 100x). Panel (**C**). Acute tubulointerstitial nephritis associated with BK virus infection. Intranuclear inclusions (*) and vacuolated cytoplasm (hematoxylin and eosin, 400×). Panel (**D**,**E**). Lymphocytic infiltration of the kidney in a patient diagnosed with marginal zone lymphoma. Infiltrating cells are CD20 positive (hematoxylin and eosin, 10×).

Anticancer drug-related nephrotoxicity is a significant adverse effect in terms of frequency and clinical relevance. Over the last few years, there has been a growth in the number of available anticancer molecules, and novel mechanisms of action are being explored. The most striking has been the enhancement of the natural immunosurveillance mechanisms of cancer, or targeted cell therapies [11]. Some of these novel mechanisms may be responsible for the appearance of ATIN. Figure 1A shows light microscopy images illustrating drug-induced acute interstitial nephritis.

### 2.1. Immune Checkpoint Inhibitors 

Immune checkpoint inhibitors (ICI) are monoclonal antibodies directed against programmed cell death-1 (PD-1), PD-1 ligand 1 (PD-L1), and cytotoxic T lymphocyte antigen 4 (CTLA4). The PD-1/PD-L1 and CTLA4 pathways are negative regulators of T cell activation and maintain self-tolerance. Cancer cells overexpress these molecules in their surface in order to inhibit attack by antitumor T cells. ICIs block these molecules, thus restoring cancer immunosurveillance mechanisms [12]. Immune-related adverse effects (irAEs) of ICI appear because of the loss of inhibition of T cells with their subsequent activation, which interferes with self-tolerance mechanisms directed to organs [13]. In kidneys, the most frequently reported irAE is ATIN, although glomerular diseases such as pauci-immune glomerulonephritis or minimal change disease have also been described during the use of ICI [14].

Early after the initiation of the widespread use of ICI, Cortazar et al. presented a series of cases that included 13 patients presenting with AKI during treatment. Renal biopsy revealed ATIN in 12 out of 13 cases, and the remaining patient was diagnosed with TMA [15]. Some authors hypothesized that ICI-driven ATIN is related to the loss of tolerance to previously tolerated drugs or renal antigens. Koda et al. reported a case of ATIN associated with ICI showing lymphocyte reactivity against esomeprazole, previously tolerated, and absence of response to nivolumab in a lymphocyte transformation test [16]. In this line, Seethapathy et al. found an increased risk of ATIN in ICI-treated patients receiving PPIs [17]. Lately, Cortazar et al. reported a 23% incidence of ATIN flares after rechallenging with ICI following a first treated and recovered ATIN episode, regardless of the current steroid dose [18]. 

Our group recently published [19] a clinical comparison between ICI-associated ATIN and ATIN related to other drugs. We found milder creatinine elevation among ICI-related ATIN cases together with a more prominent leukocyturia. Comparison of the clinical course revealed a larger latency period from drug administration to ATIN diagnosis in ICI compared to the other drugs, and a slower creatinine decrease after treatment in this cohort [1]. Preliminary unpublished data from our group suggest higher levels of inflammatory cytokines, involved in leukocyte trafficking and chemotaxis, in the urine from patients with ICI-related ATIN compared to ATIN induced by other nephrotoxic drugs. The finding of these possible urinary biomarkers argues in favor of the nature of the intrarenal inflammatory microenvironment of this entity.

### 2.2. Platinum Agents 

Platinum agents are mostly associated with tubular cell dysfunction and ATN, which are related to their uptake and accumulation in renal epithelial tubular cells [4]. Although AKI is less frequently reported during treatment with third generation platinum agents such as oxaliplatin, in comparison with cisplatin or carboplatin [20], there are several reports of ATIN during treatment with oxaliplatin. Yamada et al. reported oxaliplatin-related ATIN accompanied with autoimmune anemia and thrombocytopenia, presenting good renal and hematologic response to steroid treatment^20^. In the two cases reported by Choi et al., oxaliplatin-associated ATIN was preceded by systemic symptoms, including fever and skin rash [21]. Asai et al. reported a carboplatin-associated ATIN that was also associated with fever and malaise as in the cases reported by Choi et al. [22]. Thus, ATIN must be suspected in those individuals treated with platinum agents who developed renal impairment without recovery despite drug discontinuation, especially if systemic symptoms are present.

### 2.3. Tyrosine Kinase Inhibitors 

Tyrosine kinase inhibitors (TKIs) are effective in the treatment of various malignancies. Imatinib was the first one introduced into clinical oncology, and it was followed by gefitinib, erlotinib, sorafenib, sunitinib, and dasatinib. Their mechanism of action consists of competitive ATP inhibition at the catalytic binding site of tyrosine kinase, thus blocking the receptors of various growth factors that exert their biological effect via tyrosine kinase activation [23]. These growth factors are involved in cell proliferation and reparative processes of various epithelia such as renal tubular epithelia. Thus, TKIs can cause an AKI by interruption of these processes.

ATIN has been described relating to treatment with sunitinib [24,25] as well as sorafenib [26]. Interestingly, Khurana et al. reported the case of a patient presenting ATIN related to sunitinib administration that resolved after its withdrawal and experiencing a flare after rechallenging with a related agent, sorafenib [27]. This suggests that ATIN is a class effect within TKIs. In the setting of AKI during TKIs treatment, TMA must also be considered as a differential diagnosis [28].

### 2.4. Ifosfamide 

Ifosfamide is classified as an alkylating agent with a broad spectrum of antineoplastic activity including pediatric solid tumors, non-Hodgkin’s lymphoma, as well as acute lymphoblastic leukemia. It is a prodrug metabolized in the liver to isofosforamide mustard, the active alkylating compound [29]. Ifosfamide can cause both acute and chronic kidney injury, with a wide spectrum of clinical manifestations up to end-stage renal disease.

When ATIN occurs in the context of ifosfamide treatment, a special histological feature might be found: the presence of karyomegalic cells [30]. Karyomegalic changes in tubular epithelial cells result from aberrant cell division related to exposure to the toxic ifosfamide, secondarily inducing interstitial inflammation. By light microscopy, karyomegalic cells show enlarged hyperchromatic and irregular nuclei, with a high DNA ploidy [31]. Ifosfamide tubular toxicity is known but there are other factors, mainly genetic polymorphisms influencing drug metabolism that might increase the susceptibility to kidney damage. Several reports support the effectiveness of steroid treatment in this setting, despite its association with chronicity features [32]. 

### 2.5. Lenalidomide 

Lenalidomide is a member of a class of drugs called immunomodulatory drugs, which has emerged as a significant weapon in the arsenal of cancer therapies. It is a lead therapeutic in multiple myeloma and myelodysplastic syndromes, but also for a wide variety of neoplasms, solid or hematological. ATIN has been described in the setting of the treatment with lenalidomide [33]. Various authors reported lenalidomide-associated ATIN cases accompanied by systemic symptoms. Dihn et al. reported a case of ATIN in a patient with Hodgkin’s lymphoma treated with lenalidomide who presented concomitant skin rash [34]. Systemic symptoms associated with ATIN can be severe and include drug reaction with eosinophilia and systemic symptoms (DRESS) syndrome that can be fatal [35,36].

### 2.6. Bacillus Calmette–Guerin (BCG) 

Intravesical instillation of BCG, an attenuated variant of *Mycobacterium Bovis*, is an immunotherapeutic treatment for noninvasive bladder cancer. Attachment of the microorganism to the urothelial cells induces a local inflammatory response with infiltrating macrophages, T cells and granulocytes that eliminate the neoplastic cells [37]. Mohammed et al. published an extensive review of BCG renal toxicities. They found that the most frequent cause of AKI during BCG treatment was ATIN [38]. Due to their common histologic features, the main differential diagnosis of BCG-related ATIN is mycobacterial infection of the renal parenchyma, especially if granulomas are present. In this scenario, mycobacterial DNA detection in kidney tissue can aid in the diagnosis. Although it is considered a non-pathogenic strain, BCG can occasionally cause infection, especially in immunocompromised hosts [39,40]. Caution should be taken when systemic symptoms appear in order to differentiate from miliary tuberculosis. It is mandatory to distinguish between both entities as treatment is clearly different: corticosteroids for pure ATIN in contraposition with tuberculosis treatment. There are few reports of glomerular disease associated with BCG therapy [41,42]. Figure 1B shows interstitial necrotizing granuloma in a patient treated with BCG therapy.

### 2.7. Chimeric Antigen Receptor–Modified T Cells (CAR-T Cells)

CAR-T cells are a novel immune-mediated anticancer therapy that consists of the infusion of lymphocytes from the same patient that have been modified in vitro to recognize specific tumoral antigens regardless of their presentation through major histocompatibility complex (MHC). Once re-infused in the patients, these lymphocytes lead to an immune response against the tumor. After tumoral binding, CAR-T cells can elicit an uncontrolled inflammatory response that can lead to a cytokine release syndrome that causes hypotension and multi-organ failure [11]. In this setting, the most frequent renal lesion reported is ATN. Although CAR-T cells are engineered to specifically recognize tumoral antigens, these can also be expressed by healthy cells or found in peritumoral healthy tissue causing an on-target off-tumor infiltration and damage of non-tumoral tissue by CAR-T cells [43]. To date, to the best of our knowledge, there are no reports of this on-target off-tumor effect in the kidney. Given the fact that tumoral antigens can be retained in the kidneys during plasma clearance, one may think that the spread of the use of this therapy may cause CAR-T cell infiltration of the kidneys mimicking conventional ATIN to be reported more often in the near future. 

## 3. Infection-Associated ATIN

Infectious diseases are a recognized etiology for the development of ATIN. In turn, oncologic patients are more prone to suffer infections due to the disease itself and treatment-related immunosuppression. 

Some viral infections have been associated with ATIN in oncologic patients and especially those with hematological malignancies [44,45]. Human adenoviruses are non-capsulated double-stranded linear DNA viruses that can induce infection in both immunocompetent and immunocompromised individuals [46]. Renal involvement can manifest as interstitial nephritis with features of necrosis and cytopathic changes in the renal tubular epithelial cells (TECs). These cells can present smudge-type inclusions that are specific for adenovirus infection [44]. The adenovirus DNA can be found in renal tissue through polymerase chain reaction and adenoviral particles can be observed through electron microscopy [47]. 

The BK virus is a polyomavirus first described in 1971 in a renal transplant patient with ureter stenosis, who eliminated cells with atypical nuclear morphology in the urine. Three types of polyomavirus have since been described: BK, JC, and simian SV-40 virus. They are small, naked viruses, provided with a capsid of icosahedral symmetry, which houses a genome with a double circular DNA chain [48]. Primary infection with BK virus frequently occurs during childhood and remains latent in the kidney until reactivation, which is more frequent during immunosuppression periods [49]. In BK virus-related ATIN, TECs can present enlarged nuclei with basophilic inclusions [49]. Tubulitis is more prominent from the collecting duct to distal tubule and eosinophils may be present. Immunohistochemistry assessment is typically positive for SV-40 antigen [48,50]. Figure 1C shows ATIN in a BK virus infected patient.

Fungal granulomatous interstitial nephritis has also been described in oncologic patients [51]. In these cases, granulomatous features are frequently present and Grocott’s methenamine silver stain is useful to reveal the presence of yeast-like fungus [52]. 

## 4. Hematological Malignancies and ATIN

Hematological malignancies can be found infiltrating a variety of organs including the kidney, where they can cause or mimic AKI. Sometimes, ATIN can be the first manifestation of the disease [53,54,55]. Kidney infiltration by leukemic leukocytes or ATIN inflammatory cell infiltrates can be indistinguishable by light microscopy. In this setting, immunohistochemistry assessment is useful to define the nature of the infiltrating cells: monomorphic monoclonal in hematological malignancies vs. polymorphic polyclonal in ATIN.

ATIN can be a manifestation of a hematological disorder. Gu et al. reported a series of eight cases of light-chain–mediated ATIN, some of them as the first clinical manifestation in patients with plasma cell dyscrasia. The infiltrates were mainly composed of mononuclear cells, with the occasional presence of plasma cells and eosinophils. Ultrastructural evaluation showed subtle light chain deposition in tubular basement membrane. Features of cast nephropathy, glomerular light chain deposition disease, or amyloidosis were absent. The authors hypothesized that the presence of light chain in tubular basement membrane, mainly kappa, may alter intrinsic antigens, leading to the release of cytokines that induce the chemotaxis of inflammatory cells [56]. Other authors have also reported cases of ATIN associated with plasma cell dyscrasia [57,58,59].

Kakareko et al. reported an interesting case of ATIN coexisting with mantle cell lymphoma (MCL) infiltration. In the renal biopsy, they observed two different patterns: cellular infiltrates forming nodules that were positive for CD20, CD5, and cyclin D (MCL marker), coexisting with a diffuse interstitial infiltrate formed by CD3 positive T lymphocytes. Other causes of ATIN were excluded, and the authors suggested a link between both diseases [60]. Inoue et al. reported a patient previously diagnosed with chronic lymphocytic leukemia (CLL) presenting with AKI. Kidney biopsy revealed CD20+, CD23+, CD5−, CD10−, and BCL-2+ cells consistent with CLL origin, coexisting with CD3+/CD5+ T cells that formed nodular granulomas indicative of ATIN infiltrates, as in the patient reported by Kamat et al. [61]. Figure 1D,E show CD20 positive tubulointerstitial lymphocytic infiltrate in a patient diagnosed with marginal zone lymphoma.

Acute graft versus host disease (GVHD) is a complication of hematopoietic stem cell transplantation (HST). It is the second leading cause of mortality after HST. Antigen presenting cells from the recipient expose alloantigens that are recognized by the infused donor-T cells, thus becoming activated, proliferating, and producing cytokines that amplify the inflammatory response affecting multiple organs [62]. GVHD can present itself as an AKI, with different underlying pathological features, including ATIN [63]. GVHD manifests in other organs with infiltration of lymphocytes; thus, in this setting, ATIN may be related to the infiltration of donor lymphocytes into the recipient’s kidney [64]. Homma et al. reported a patient who suffered GVHD after HST in the context of acute myeloid leukemia treatment. GVHD debuted with gastrointestinal symptoms and progressive renal failure. Kidney biopsy revealed tubulitis with tubular epithelial vacuolization with unaltered glomeruli [65]. Kusumi et al. reported autopsy findings of 26 allo-HST recipients who had GVHD without AKI. They identified the presence of tubulitis in 3 out of 26 individuals [66]. El-Seisi et al. reported that 67% of patients had tubulitis (16/24) in their 24 autopsies with GVHD and renal failure. Tubulitis was one of the most frequent histological features in GVHD associated with kidney dysfunction, together with other tubular epithelial abnormalities such as tubular epithelial atypia and tubular calcification [64]. 

## 5. Conclusions

ATIN is a frequent cause of AKI in oncological patients. It can occur as an adverse effect of some anticancer drugs or other concomitant medications required by these patients. Other less frequent etiologies must be considered when evaluating ATIN in this population, such as atypical presentation of viral or fungal infections due to immunosuppression related to cancer. Finally, the differential diagnosis of ATIN with hematological malignancies should be considered due to the similarity of the diseases under the light microscope and the fact that renal failure may be the first manifestation of a hematological disease. 

## Data Availability

Not applicable.
